# Capacitance of Membrane As a Prognostic Indicator of Survival in Head and Neck Cancer

**DOI:** 10.1371/journal.pone.0165809

**Published:** 2016-11-01

**Authors:** Teresa Małecka-Massalska, Radosław Mlak, Agata Smoleń, Anna Brzozowska, Wojciech Surtel, Kamal Morshed

**Affiliations:** 1 Human Physiology Department, Medical University of Lublin, Lublin, Poland; 2 Department of Epidemiology and Methodology of Clinical Research, Medical University of Lublin, Lublin, Poland; 3 Oncology Department, Medical University of Lublin, Lublin, Poland; 4 Institute of Electronics and Information Technology, Technical University of Lublin, Lublin, Poland; Taipei Medical University, TAIWAN

## Abstract

**Background:**

Evaluation of prognostic value of capacitance of membrane (Cm), parameter measured by bioelectrical impedance (BIA) as an alternative to known clinical factors in patients with Head and Neck Cancer (HNC).

**Methods:**

A cohort of 75 stage IIIB and IV HNC patients treated in Department of Otolaryngology, Head and Neck Surgery, Medical University of Lublin, Poland were prospectively evaluated. Cm measurements were performed in all patients using a bioelectrical impedance analyzer that was set on a frequency of 50 kHz. Results of Cm measurements were presented in nF. Survival differences were estimated using Kaplan–Meier method.

**Results:**

Significantly higher Cm median was noted in well-nourished(n = 45) compared to malnourished (n = 30) patients (1.41 vs 1.01 respectively; p = 0.0009). Established in ROC curves analysis cut-off value (0.743) was characterized by 98% specificity and 37% sensitivity in the detection of malnutrition. Median overall survival (mOS) in the cohort was 32months. At the time of analysis deaths were recorded in 47 cases (62.7%). In patients who had Cm below the level of 0.743 risk of OS shortening was significantly higher than in other patients (12.1 and 43.4 months respectively; HR = 8.47, 95%CI: 2.91–24.66; χ^2^ = 15.38, p = 0.0001).

**Conclusion:**

Cm is a strong, independent prognostic factor in head and neck cancer.

## Introduction

The idea of classifying cancers based on their electrical properties has a long story. It was proposed by Fricke and Morse in 1926 [1]. The electrical properties of cancer cells are different than the electrical properties of the normal tissues that surround them. Cancer cells have higher intracellular sodium, lower intracellular potassium, magnesium and calcium concentrations, and more negative charges on their cell surface. These abnormalities result in cancer cells having lower transmembrane potentials and electrical impedance than normal cells and altered membrane permeability [2,3].One of the first researchers, who noticed that biochemical explanations alone fail to explain the role of electricity in cellular regulation was Szent-Gyorgyi. This well recognized scientist and Nobel prize laureate believed, that the cells of the body possess electrical mechanisms and use electricity to regulate and control the transduction of chemical energy and other life processes. Other investigators believe, that electrochemical forces across the membrane regulate chemical exchange across the cell. They also claims, that electrical changes may precede biochemical disorders and thus also clinical symptoms [4,5].Bioelectrical impedance analysis (BIA)assesses body properties e.g.: reactance (Xc) and resistance (R) by recording voltage change in applied current [6]. The relationship between resistance and reactance is described by a calculated parameter which is phase angle (PA). The formula for PA is: Phase angle = arc – tangent reactance/resistance x 180°/π. PA reflects the relative contributions of fluid (resistance) and cellular membranes (reactance) of the human body. By formula, PA is positively associated with reactance and negatively with resistance [6]. Another raw parameter which is derived from BIA is capacitance of membrane (Cm). Cm is considered to be a physical quantity equal to the ratio of charge collected on the conductor to the potential of the conductor. Reactance is associated to the capacitance properties of the cell membrane, and its alterations can be determined by change of the composition, integrity and function of this structure (Reactance = 1/2 × π × frequency × Capacitance) [7, 8]. In biological systems the membrane behaves as capacitor when exposed to the alternating current. The ion concentration gradient across that membrane causes the electrical potential. If there is no “electricity”, the cell is damaged. Cm somehow describes “how much of oscillating current” caused by electric ion flow is across the cell membrane. Cm can be calculated from the formula which takes into account resistance at 0 and infinite frequencies and the characteristic frequency of maximal reactance [9]. BIA is well established tool of objective evaluation of body composition and thus nutritional status in different diseases such as cancer [10–13]. The utility of these tools has been assessed by their ability to predict different clinical outcomes such as: treatment response, complications, quality of life (QoL) and survival [13,14].Many BIA parameters were compared between each other to evaluate ability of prediction of different clinical outcomes, but only few evaluate ability to predict overall survival [14].To date there are no studies evaluating Cm value as prognostic indicator in HNC. This prospective study was conducted to investigate the impact of Cm on patient survival and to identify prognostic utility of this tool in well-nourished and malnourished (according to Subjective Global Assessment Scale; SGA) adult patients with HNC.

## Materials and Methods

All procedures performed in this study involving human participants were in accordance with the ethical standards of the institutional and/or national research committee and with the 1964 Helsinki declaration and its later amendments or comparable ethical standards. Research Ethics Committee of the Medical University of Lublin, Poland approved this study (consent no.: KE-0254/170/2009). This article does not contain any studies with animals performed by any of the authors. Informed consent was obtained from all individual participants included in the study.

Study group consist of 75 patients prepared for surgical operation due to HNC. All patients were treated at the Otolaryngology Department, Head and Neck Surgery, Medical University of Lublin, Poland between October 2009 and October 2012.Study enrollment criteria:(a) at least 18 years old (b) histologically confirmed diagnosis of primary squamous cell carcinoma of HNC (in the absence of other cancers), (c) lack of prior cancer treatment (chemotherapy, radiotherapy, molecularly targeted therapy), (d), obtain of informed consent before study entry, (e) appropriate laboratory results: renal function (Creatinine clearance ≥50 ml/minute), liver function, complete blood count (f) lack of metallic implants, (m) the presence of all limbs, (g) absence of cardioverter or defibrillator.

### Outcome measures

In all patients detailed evaluation, including demographic(sex, age), tumor (type, stage, grade, size and site), clinical (metastases, symptoms and comorbidities) and nutritional related data (laboratory tests: albumin, total protein, transferrin; SGA—established by a medical doctor before hospitalization starts; BIA) was performed. SGA assessment covered: physical examination (low levels of subcutaneous muscle and fat mass, ascites, sacral or ankle edema) change in weight, dietary intake, gastrointestinal symptoms. Results of SGA were identified as normal (0), mild (1+), moderate (2+), or severe (3+).[15]. Patient's nutritional status was defined as SGA-A (well-nourished), SGA-B (moderately malnourished) or SGA-C (severely malnourished) based on physical examination (evaluation of losing of subcutaneous fat, muscle wasting, presence of ankle and sacral edema and ascites) and medical documentation. Every time prior to consultation, a physician reviewed the patient's medical record and verified any change in patient's weight. Subsequently, patient under physician supervision reviewed the Patient-Generated Subjective Global Assessment (PG SGA) form to obtain answers to all the questions. BIA was conducted using Impedi Med bioimpedance analysis SFB7 BioImp v1.55 (Pinkenba Qld 4008, Australia). During BIA patients were lying supine, their legs and arms were not touching the torso. All measurements were performed on the patients’ right side. The four surface standard tetra polar electrodes technique on the foot and hand was used. R and Xc were measured three times in each patient (mean values were than calculated), directly in Ω at 50 kHz. Cm values were automatically obtained from the equipment.

### Statistical analysis

The statistical calculations were performed using Statistica8.0 (StatSoft) and MedCalc 10 (MedCalc Software) computer software. Overall survival was defined as the time from diagnosis to death (complete data), or to the last recorded visit, last contact or last known to be alive (censored data). To illustrate the differences in survival Kaplan-Meier estimation method was used. The log rank test was used to compare the survival distribution according to different variables. The difference was considered to be statistically significant if p≤0.05. For survival analysis, Cm results were categorized into two groups according to its median, which was equal 0.743(cut-off value*). Above value was established based on ROC curve analysis. In Cox regression analysis, the following variables were included: age at diagnosis, sex, Cm, SGA, serum transferrin and albumin. Variables in the multivariate Cox model were selected with the backwarde limination likelihood ratio method and with thresholds of<0.05 for entry and>0.10 for removal of variables. For Cox regression analysis, the Cm data were categorized in the same way as for Kaplan-Meier estimation.

Cm was the main measure for determining the sample size. The sample size was estimated on the basis of the pilot study results in the control group and the study group (N = 31 in each group) using Altman nomogram. Assuming the test power for two independent, equally numerous groups to be at least 80% and obtaining standardized difference in the Cm of 0.45 at 5% level of statistical significance, the estimated required size of each sample was 75 cases.

## Results

In the study group there was 8 women and 67 men. Median age of patients was 56 years. All patients have histologically confirmed HNC (localizations of tumors: larynx—28, middle pharynx—21, oral cavity—18, inferior pharynx—8) All neoplasms were squamous cell carcinomas (SCC). Baseline characteristics of the study group and evaluated parameters were presented in Table 1.

**Table 1:** Baseline characteristic of patients with a new diagnosis of head-and-neck cancer (N = 75).

Characteristic	Number	Percent (%)
**Sex**		
Male	67	89.3
Female	8	10.7
**Prior treatment history**		
Newly diagnosed	75	100
**Tumor stage at Diagnosis**		
Stage III	27	36
Stage IV	48	64
**Nodal stage**		
N0	27	36
N1	17	22.7
N2	26	34.7
N3	5	6.6
**Subjective Global Assessment**		
Well-nourished (SGA A)	45	60
Moderately malnourished (SGA B)	24	32
Severely malnourished (SGA C)	6	8

**Fig 1:**
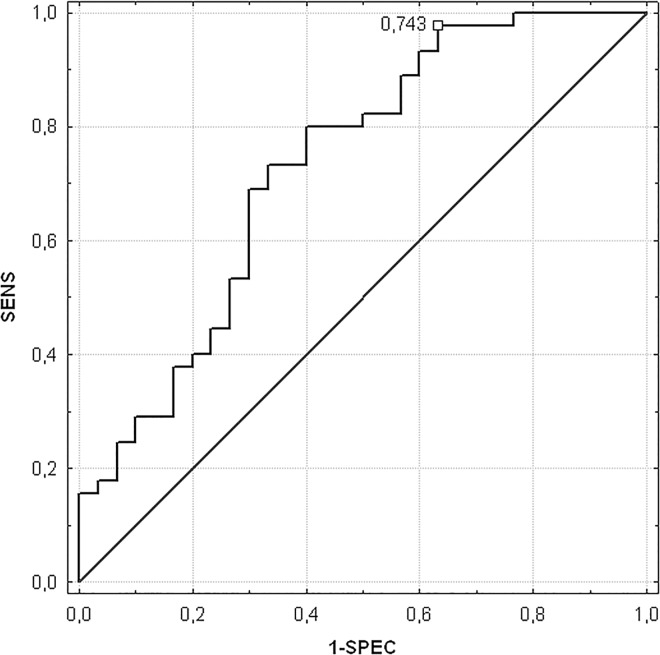
A receiver operating characteristic curie assessing an optima cut-off point Cm.

According to SGA scale 60% of patients were well-nourished, whereas 40% was classified as moderately or severely malnourished. In well-nourished group of patients, value of Cm was significantly higher (1.41±0.50 vs. 1.01±0.43, respectively; p = 0.0009) compared to moderately or severely malnourished. The optimal Cm cut-off value of 0.743estimated by ROC curve analysis was characterized by 98% sensitivity and 37% specificity. Therefore, Cm value provides modest diagnostic accuracy to distinguish well-nourished and malnourished status (p = 0.0009; AUC = 0.7, 95% CI = 0.61–0.85). ROC curve analysis for Cm was shown in Fig 1.

Baseline characteristics of evaluated parameters were presented in Table 2. The distribution of Cm value (< or ≥ 0.743) did not depend on demographic and clinical factors such as: gender, location of tumor and stage of disease. However Cm value significantly depend on age. Cm value above the level of 0.743were significantly more frequent recorded in older patients (≥55 years). Distribution of Cm value according to demographic and clinical factors were shown in Table 3.

**Table 2:** Assessment of baseline characteristics in 75 patients by Cm ratio (N = 75).

Characteristic	Mean	Standard Deviation	Range	*P*, *Z*
**Age at diagnosis (years)**	56.88	8.21	37–80	-
**Total protein (mg/dl)**	7.14	0.57	5.50–8.30	
**Albumin (g/dl)**	4.03	0.37	3.10–4.70	
**Transferrin (mg/dl)**	202.47	39.63	140–312	
**Cm overall (nF)**	1.75	0.55	0.71–3.24	
**Cm (SGA A) (nF)**	1.41	0.50	0.62 – 2.86	**0.0009** 3.33
**Cm (SGA B+C) (nF)**	1.01	0.43	0.40 – 1.94	

**Table 3:** Distribution of Cm value according to demographic and clinical factors.

Variable	Cm*		*p*, χ^2^
	< 0.743	≥ 0.743	
	12 (16%)	63 (84%)	
**Gender**			
Male	11 (16.2%)	57 (83.8%)	0.680 0.169
Female	1 (14.3%)	6 (85.7%)	
**Age** (years)			
<55	1 (2.7%)	36 (97.3%)	**0.005** 7.754
≥ 55	11 (29%)	27 (71%)	
**Location of tumor**			
Upper: mouth, tongue, jaw, tonsil, nose, center throat, maxillary sinus.	5 (18.5%)	22 (81.5%)	0.804 0.061
Lower: larynx, glottis, lower part of the throat.	4 (20%)	16 (80%)	
**Stage of disease**			
IIIB	3 (13%)	20 (87%)	0.87560.025
IV	9 (17.6%)	42 (82.4%)	

* Threshold value determined using the ROC curve analysis.

**Fig 2:**
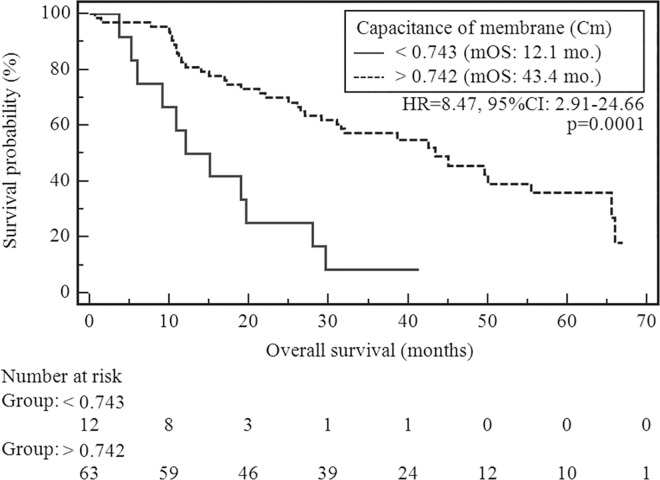
The probability of overall survival change depending on Cm value.

Median overall survival (mOS) in the study group was 32 months. At the time of analysis deaths was recorded in 47 cases (62.7%). In patients who had Cm value below level of 0.743 risk of OS shortening was significantly higher than in other patients (HR = 8.47, 95%CI: 2.91–24.66; χ^2^ = 15.38, p = 0.0001).OS in group with Cm below and above level of 0.743 were 12.1 and 43.4 months respectively. The survival curves stratified by Cm value was presented in Fig 2. In the case of other demographic and clinical factors (gender, age, tumor localization and stage of disease) there were no statistically significant differences in the duration of OS in the study group. Univariate analysis of demographic and clinical factors was demonstrated in Table 4.

**Table 4:** Univariate Kaplan-Meier survival analysis.

Variable	Statistical significance: *P*, χ^2^	HR (95% CI)
**Gender**	0.8327, 0.0446	1.1120 (0.4153–2.9770)
Male (M)		
Female (F)		
**Age**	0.5476, 0.3616	0.8386 (0.4724–1.4885)
≥55		
<55		
**Location of tumor**	0.9890, 0.0002	1.0050 (0.4946–2.0422)
Upper: mouth, tongue, jaw, tonsil, nose, center throat, maxillary sinus.		
Lower: larynx, glottis, lower part of the throat.		
**Stage of disease**	0.9798, 0.0006	0.9920 (0.5345–1.8414)
IIIB		
IV		
Cm*	**0.0001,** 15.3802	8.4734 (2.9123–24.6537)
**<**0.743		
**≥** 0.743		

* Threshold value determined using the ROC curve analysis.

Cox multivariate logistic regression demonstrated, that only Cm value (HR = 3.73, 95%CI: 1.45–9.61; p = 0.0065) was independent prognostic factor for OS in the study group (overall model fit:χ^2^ = 9,085, p = 0.1057). Results of multivariate Cox regression analysis was presented in Table 5.

**Table 5:** Multivariate Cox proportional hazards model.

Variable	β coefficient	*P* value	HR(95% CI)
**Gender**	0.3383	0.5995	1.4025 (0.3994–4.9252)
**Age**	0.2375	0.5794	1.2681 (0.5498–2.9249)
**Location of tumor**	0.0259	0.9459	0.9744 (0.4624–2.0533)
**Stage of disease**	0.2012	0.6253	1.2229 (0.5476–2.7311)
Cm*	1.3176	**0.0006**	3.7341 (1.4522–9.6061)

* Threshold value determined using the ROC curve analysis.

## Discussion

Evolving weight loss and malnutrition frequently occur in cancer patients, including head and neck, gastrointestinal and lung cancer [16–18]. Weight loss during treatment for HNC is a major concern. It must be underlined that there is no consistent objective tool for nutrition diagnosis in oncology. The topic is complicated by the lack of universal agreement on the operational definition of malnutrition and on the validity of the assessment indicators. In the clinical practice the most common method by which the nutrition assessment is performed is the SGA. The SGA is well validated subjective tool and most frequently used technique of nutritional assessment in cancer patients [17]. The SGA is a clinical technique that combines data from subjective and objective aspects of medical history if they are available. Currently, most of the nutrition screening in oncology settings is completed by doctors or nursing professionals. Another method used for nutrition state is BIA, which is an objective and useful nutritional diagnostic method for health but also for chronic diseases (amyotrophic lateral sclerosis [10], cancer [19], cirrhosis [20], hemodialysis [21], HIV [22,23]). The raw parameters of BIA derived from reactance and resistance are the fat mass, the fat-free mass, the total body water, the extracellular water content, the intracellular water content. BIA measures reactance (Xc) and resistance (R) by registering voltage drop in distributed current [6]. Resistance is the impediment to the flow of the current, related to the extent of water present in the body. Reactance is the restriction produced by the cell membranes and tissue interfaces [24]. Reactance makes the current delay the voltage generating a phase shift, or phase angle, which is the ratio of reactance to resistance. We suggest, that raw data derived from BIA (resistance, reactance) correspond almost directly with the status of nutrition state described by SGA. Resistance and reactance are needed to obtain PA which correlates with the nutrition stage (Cm is also calculated from reactance). In 2006 De Luis DA et al. in a case-control study investigated the utility of impedance parameters in a population of 67 males with HNC [21]. They found that in cancer patients, reactance (62.3 +/- 17.2 vs. 56.6 +/- 15.1 ohm; p < 0.05) and PA (8.02 +/- 1.3 vs. 6.9 +/- 1.5 degrees; p < 0.05) were lower than in control healthy patients [25]. On the other hand in our study we observed smaller distribution of water among the extra- and intracellular space. Moreover there was a higher resistance of the electric current because of the smaller distribution of water, (resistance was lower (p = 0.0002) in the control group compared to HNC patients (513.73 ± 65.79 ohm vs 596.24 ± 96.31 ohm, respectively). However, the difference in reactance between two groups (HNC and control) was not found [26].In our another study on HNC patients we investigated whether there are any tissue electrical differences before/after surgery treatment in patients with HNC. Resistance was significantly (p = 0.0005) higher after than before surgery in patients with HNC (596.24 ± 96.31 ohm vs 647.64 ± 276.11 ohm, respectively).PA (construed as a ratio of resistance and reactance measured at 50 kHz was significantly (p = 0.000009) lower after than before surgery in patients with HNC (4.69° ± 0.71vs 4.22 ± 0.83, respectively) [27].

Since a long time it has been known that the electrical properties of cancer cells are different than the electrical properties of the normal tissues that surround them [2,3].Based on that information we went with our hypothesis further and tried to find the differences in electrical tissue properties between well-nourished cancer patients and those who were malnourished or cachectic. Additionally, the impact of the selected parameter which was Cm on patient survival was investigated. To date, this study is the first which evaluates such utility of Cm (parameter obtained directly from the device, thus objective).Although Cm parameter has not been previously evaluated in the context of the OS, an impact of other BIA parameters (especially PA) on survival has been already described. In various cancers, lower PA values were associated with significantly lower survival rates [14,28,29–31]. In study Hui et al. it was shown that PA is a relevant and independent of established prognostic factors predictor of poor survival in advanced cancer patients[32]. In cited study collective results for general population of advanced cancer (gastrointestinal, breast, genitourinary, gynecological, hematological, respiratory, head and neck and others) were shown. The key issues of prognosis in HNC patients are stage of disease and tumor grade but previous treatment has no impact on patients within this study because only treatment naïve hospitalized patients with III or IV stage were included. Findings of Hui et al. 2014 and Davis et al. 2009 researches confirmed that low PA values discriminate the patients with short life expectancy, whereas higher PA is correlated with improvement of survival [32,33].Our study for the first time shows, that another parameter of BIA namely Cm is beside many other known prognostic factors (albumin, transferrin) an independent prognostic factor in advanced HNC. However, the current study has several limitations. First, we enrolled only small population of patients in advanced HNC hospitalized in a single specialized center. Further research is necessary to determine whether the current study findings also apply to all the advanced patients also in the outpatient care or generally in advanced HNC.

## Conclusion

Cm is a significant, independent of established prognostic factors predictor of survival in patients with advanced HNC.
